# Antitumor and Antiangiogenic Effect of Tannic Acid in the Advanced Stage of Ehrlich Ascites Tumor in Mice

**DOI:** 10.3390/ijms26189070

**Published:** 2025-09-17

**Authors:** Nada Oršolić, Martina Kunštić, Maja Jazvinšćak Jembrek

**Affiliations:** 1 Division of Animal Physiology, Faculty of Science, University of Zagreb, Rooseveltov trg 6, 10000 Zagreb, Croatia; martina.kunstic@gmail.com; 2 Division of Molecular Medicine, Ruđer Bošković Institute, Bijenička Cesta 54, 10000 Zagreb, Croatia; maja.jazvinscak.jembrek@irb.hr; 3 School of Medicine, Catholic University of Croatia, Ilica 244, 10000 Zagreb, Croatia

**Keywords:** tannic acid, mice, Ehrlich ascites tumor, peritoneal angiogenesis, cyclooxygenase-2 inhibition

## Abstract

Ehrlich ascites tumor (EAT) is a rapidly growing, angiogenesis-dependent tumor characterized by high levels of vascular endothelial growth factor (VEGF). VEGF contributes to ascites formation, which supports tumor cell growth and the accumulation of tumor-associated macrophages (TAMs), primarily of the immunosuppressive M2 phenotype. M2 macrophages promote tumor progression by secreting angiogenic and immunomodulatory factors such as VEGF, matrix metalloproteinases (MMPs), and cyclooxygenase-2 (COX-2). This study investigated the effects of tannic acid (TA) on tumor growth and angiogenesis in EAT-bearing mice, focusing on TAM–tumor cell interactions. We evaluated ascites volume, cell counts, macrophage activity, peritoneal angiogenesis and blood vessel density, concentrations of VEGF, COX-2, and MMP-2/-9, blood biomarkers, and DNA damage using the comet assay. TA treatment significantly reduced tumor growth and angiogenesis by modulating TAM function. Specifically, TA inhibited VEGF, COX-2, and MMP-2/-9 expression, decreased M2 macrophage numbers, and enhanced the antitumor immune response, as shown by increased lymphocyte activation and favorable shifts in lymphocyte-to-monocyte (LMR) and neutrophil-to-lymphocyte (NLR) ratios. Additionally, TA induced DNA fragmentation in tumor and blood cells, indicating cytotoxicity and potential induction of apoptosis. These findings suggest that TA’s inhibition of TAMs may be a promising strategy for treating tumors and other angiogenesis-related conditions.

## 1. Introduction

Malignant ascites, the abnormal accumulation of fluid in the peritoneal cavity, is a common clinical sign of abdominal cancers. At least three different pathological mechanisms can cause ascites formation: (i) decreased lymphatic drainage from the peritoneal cavity due to obstruction of lymphatic vessels by tumor cells; (ii) increased permeability of the peritoneal capillaries; and (iii) enhanced angiogenesis [1,2]. Among numerous factors, vascular endothelial growth factor (VGEF), which increases vascular permeability and promotes the growth of new blood vessels, has been recognized as a major contributor to ascites formation.

The presence of malignant ascites usually indicates tumor progression, which is influenced by numerous factors, including genetic and epigenetic modifications, oxidative stress and accumulation of reactive oxygen species (ROS), and impaired immune surveillance [2,3]. Moreover, tumor actively recruits monocytes and macrophages from the circulation and promotes their differentiation into tumor-associated macrophages (TAMs). Stimulated by various signaling molecules and stromal cells, these macrophages undergo phenotypic and functional changes that change tumor microenvironment and accelerate tumor growth. In particular, macrophages shift from the classical M1 phenotype to the alternative M2 phenotypes. While M1 macrophages exhibit pro-inflammatory and antitumor activities, M2 macrophages promote immune suppression and tumor cell proliferation. M2-polarized TAMs play a crucial role in tumor progression through the several mechanisms: (i) immunosuppression and release of anti-inflammatory cytokines; (ii) shaping of tumor-promoting microenvironment through the production of survival/growth factors, such as VEGF; (iii) release of proangiogenic factors (MMP-2/-9 and COX-2); and (iv) increase in the activity of arginase (Arg) and decrease in the activity of inducible nitric oxide synthase (iNOS). Moreover, these M2 macrophages are strongly related to poor prognosis of cancer patients [2,3,4,5]. They have weak antigen-presenting capabilities, and through the secretion of various suppressive factors, they reduce the activity of cytotoxic T cells and natural killer (NK) cells [2,3,4,5]. Consequently, targeting TAMs is considered a promising new approach in cancer therapy [2,3,4,5]. In addition, as evidence indicates that COX-2 is the key enzyme involved in M2 polarization [3,5], it has been suggested that COX-2 inhibition may repolarize TAMs toward the M1 phenotype and restore antitumor immunity [2].

Epidemiological data indicate that diets rich in fruits and plants with high content of polyphenolic antioxidants are associated with a lower incidence of cancer [6,7,8,9,10]. Phenolic compounds, including tannins, are commonly found in plants. They play protective roles in animals and humans due to their antimicrobial, antiviral, antifungal, anesthetic, antiprotozoal, anti-inflammatory, antioxidant, antitumor and immunomodulatory properties. Tannic acid (TA), the specific form of tannin approved by the US Food and Drug Administration (FDA), is a naturally derived, water-soluble polyphenol obtained from various plants and beverages, such as grapes, red wine, coffee, nuts, beans, and green tea. Structurally, TA (penta-m-digalloyl glucose) is hydrolyzable tannin that contains glucose moiety core [6]. The hydroxyl groups of this glucose are esterified with five digallic acids. Studies suggest that TA can act as both an antioxidant or prooxidant, with its prooxidant activity promoting DNA damage and cell death through apoptosis [11,12,13,14,15,16]. Furthermore, non-specific activation of macrophages by TA contributes to direct cytotoxic effects on tumor cells, tumor cell damage, inhibiting angiogenesis and inducing apoptosis [6,17,18].

The anticancer potential of TA has been demonstrated in numerous tumor models in vivo and in vitro [6, 8,19–21]. TA suppressed the growth of skin, lung and stomach tumors induced by polycyclic aromatic hydrocarbons and N-methyl-N-nitrosourea in mice [6,8,19,20,21]. Several studies have revealed its chemopreventive properties. Thus, TA inhibited UV-B-induced skin tumor promotion in hairless mice by 70% [22]. Similarly, low doses of TA induced a potent dose-dependent chemotherapeutic effect against spontaneous liver tumor in male C3H mice (reducing tumor development by 87%) [23]. Another study has shown that TA increased the survival rate of tumor-bearing BALB/c mice by 30% [24].

The molecular mechanisms underlying antitumor activity of TA, including effects on malignant ascites, remain insufficiently understood. The aim of the study was to investigate the effects of TA on EAT growth and angiogenesis in Swiss albino mice. EAT is a well-established model for studying the interplay between the immune system, oxidative stress, angiogenesis, and tumor progression. EAT is fast growing tumor that is dependent on neoangiogenesis and high levels of VEGF. Its growth is further promoted by TAMs of the M2 phenotype, which are abundantly present in ascites within the peritoneal cavity. We hypothesize that TA may induce TAM repolarization and reduce VEGF/COX-2/MMP levels, and consequently inhibit angiogenesis and tumor growth. Therefore, to provide deeper insight into the effects of TA on tumor growth, angiogenesis and immunomodulation, we evaluated blood biomarkers, macrophage polarization and their interconnections with VEGF, MMP-2/-9, COX-2, microvessel density, and cytokine levels. In addition, we examined the potential pro-oxidant effects of TA and the resultant oxidative stress-induced DNA damage in tumor cells, using the alkaline comet assay.

## 2. Results

### 2.1. Change in Tumor Weight of Animals During the Experiment

EAT is a spontaneous murine mammary adenocarcinoma characterized by rapid, undifferentiated proliferation, and a pronounced increase in ascites [25,26]. In addition, EAT is highly responsive to various forms of tumor therapy, including phytotherapy. Considering these characteristics, monitoring changes in body weight serves as a valuable parameter for assessing the effectiveness of TA treatment.

**Figure 1:**
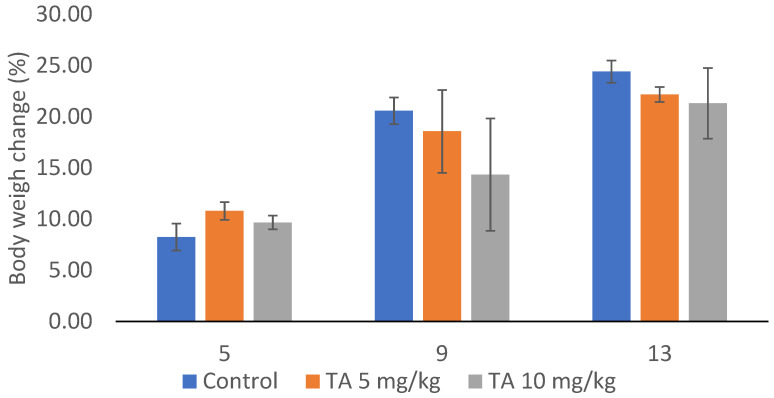
Changes in tumor weight of mice bearing EAT and treated with tannic acid. Mice (*n* = 7 per group) were injected intraperitoneally (*ip*) with 2.5 × 10^6^ viable EAT cells and treated with TA at doses of 5 and 10 mg/kg *ip* during the exponential tumor growth phase on days 5, 7, 9, and 11. Tumor growth is expressed as a percentage relative to the initial body weight of each mouse within the group.

At the beginning of the experiment, there were no significant differences in body weight among the animals across all groups. Interestingly, body weight did not change significantly throughout the experiment. The percentage of weight change relative to the initial body weight in the TA-treated groups at doses 5 or 10 mg/kg was 22.17 ± 0.73 and 21.30 ± 3.45%, respectively, compared to 24.40 ± 1.08 in the control group (Figure 1).

### 2.2. TA Inhibits Total Cell Number and Tumor Growth in Mice Bearing EAT

As a rapidly growing tumor, EAT induces a pronounced local inflammatory reaction, forms edema, and promotes cell migration and progressive formation of ascitic fluid. The fluid serves as a nutritional source that supports growth and proliferation of tumor cells and peritoneal macrophages. In this study, we focused on the collection of ascitic fluid from the abdominal cavity and analyzed its volume and total number of cells present. While TA treatment did not significantly reduce the volume of ascitic fluid compared to the control group (Table 1), likely due to its astringent and fixative-like properties, it markedly reduced the total number of cells in the peritoneal cavity. The total number of cells was 223.98 ± 27.73 × 10^6^ or 223.14 ± 28.40 × 10^6^ following treatment with 5 or 10 mg/kg of TA, respectively, compared to 891.78 ± 76.32 × 10^6^ (*p* < 0.01) in the control group. Therefore, TA at both doses inhibited tumor growth by approximately 75%.

**Figure 2:**
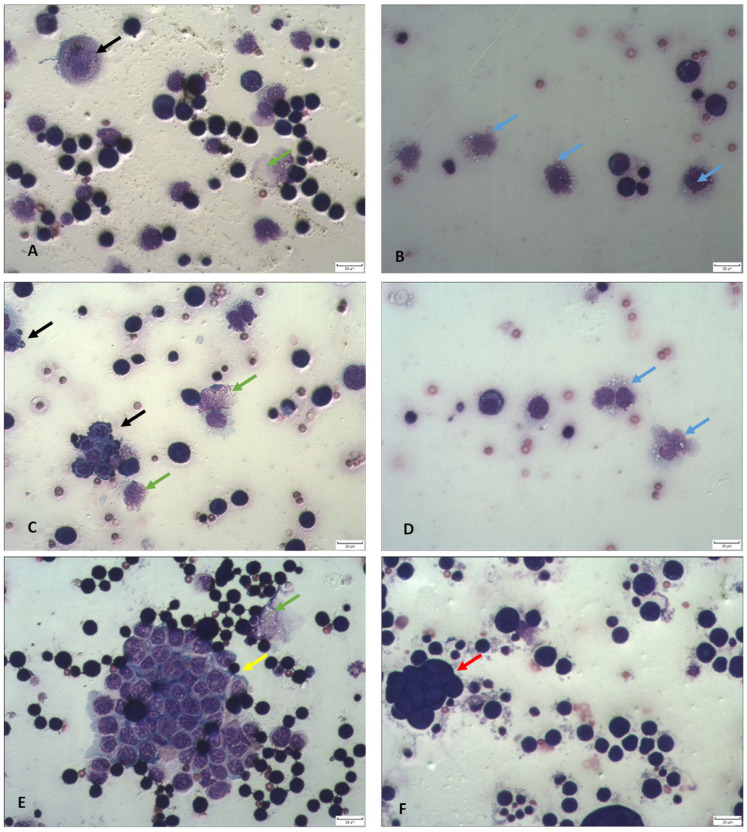
TA affected cell survival by inducing apoptosis and necrosis. (**A**,**B**) Mice bearing EAT treated with TA at a dose of 5 mg/kg. (**C**–**E**) Mice bearing EAT treated with TA at a dose of 10 mg/kg. (**F**) Control mice bearing EAT treated with saline. Morphological changes induced by TA were observed in Giemsa-stained preparations. Blue arrows indicate activated macrophages; black arrows indicate condensed and fragmented nuclei and formation of apoptotic bodies; yellow arrows show cell disintegration due to membrane disruption, green arrows show lysed cells; and red arrows show accumulation of tumor cells. The cell preparations were photographed under a light microscope at ×400 magnification (scale bar = 20 µm).

TA appears to exert its antitumor effects through the induction of apoptosis and necrosis (Figure 2). Notably, peritoneal ascites smears from TA-treated mice have revealed a high number of EAT cells with hallmarks of apoptosis, including nuclear condensation, fragmentation, cell membrane blebbing and the presence of apoptotic bodies (Figure 2). In addition, an increase in microvilli and cytoplasmic bleb formation was also observed, which may reflect the fixative-like properties of TA on EAT cells in suspension.

### 2.3. Differential Analysis of Ascites Cells in Peritoneal Lavage Fluid and Peripheral Blood Count

Differential analysis of ascites cells in the peritoneal lavage fluid showed that TA reduces the number of tumor cells at both doses tested. In addition, TA treatment led to a slight increase in lymphocyte numbers and significant changes in the proportions of neutrophils and macrophages (Table 2). The neutrophil-to-lymphocyte ratio (NLR) and lymphocyte-to-monocyte ratio (LMR) are blood-based markers that can be used as indicators of systemic inflammation and the host immune response, both of which are closely linked to cancer progression [27]. As shown in Table 3, mice treated with TA at a dose of 10 mg/kg had a significantly higher number of leukocytes in the blood (*p* < 0.01), while the differential blood analysis revealed a markedly increased number of lymphocytes compared to the control group (2.88 ± 0.53 vs. 0.76 ± 0.15).

The ratio of polymorphonuclear to mononuclear (P/M) cells indicated polymorphonuclear dominance in mice treated with 5 mg/kg TA, while in the 10 mg/kg TA group, the numbers of polymorphonuclear and mononuclear cells were similar. Interestingly, analysis of the absolute NLR and P/M ratios showed that NLR in the control group was approximately 2.8-fold higher compared to TA-treated mice (5 and 10 mg/kg). Similarly, the P/M ratio was 2.88 and 2.24 times higher in control mice compared to those treated with TA at 5 mg/kg and 10 mg/kg, respectively. The LMR was the highest in TA-treated mice at a dose of 10 mg/kg (LMR = 7.82), more than three times higher compared to the control group (LMR = 2.41).

### 2.4. TA Affects Functional Activity and Macrophage Polarization

Functional analysis of macrophages showed an increased number of active, large adherent cells with irregular nucleolar contours, abundant cytoplasmic vacuoles, and extended plasma membrane projections. These morphological changes indicate that TA has the ability to activate macrophages (Figure 2). The macrophage spreading index in the peritoneal cavity of animals treated with TA at doses of 5 or 10 mg/kg was 89.75% and 91.56%, respectively, compared to 29.63% in the control group.

**Figure 3:**
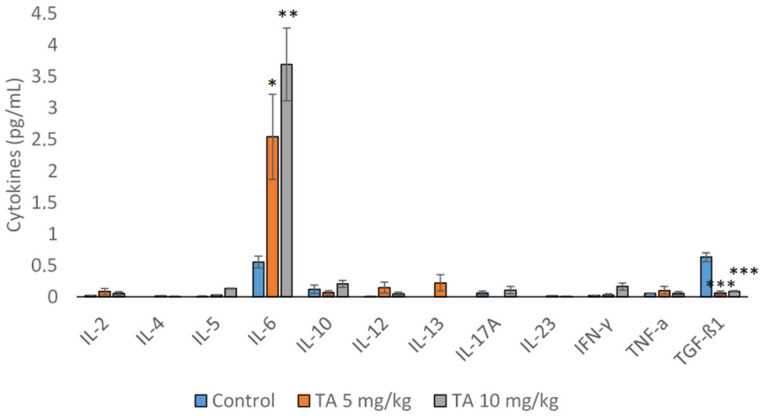
Th1, Th2 and Th17 cytokine levels in the ascitic fluid of EAT-bearing mice treated with tannic acid. Mice were injected intraperitoneally (*ip*) with 2.5 × 10^6^ viable EAT cells and treated with TA at doses of 5 and 10 mg/kg *ip* during exponential tumor growth phase on days 5, 7, 9, and 11. On day 13, mice were sacrificed and cytokine levels in the ascitic fluid were estimated by ELISA. The results are expressed as the mean ± SE of the mean from three independent experiments; * *p* < 0.05; ** *p* < 0.01; *** *p* < 0.001 compared to control group. Abbreviations: EAT, Ehrlich ascites tumor; TA, tannic acid; SE, standard error.

Functional differences of macrophages are reflected in their metabolism: M1 macrophages metabolize arginine into NO, the cytotoxic molecule, while M2 macrophages convert arginine into ornithine, which promotes tissue repair [2,3,7]. Hence, to determine whether the macrophages induced by TA were polarized toward M1 or M2 phenotype, we measured levels of NO and arginase-1 (Arg1) in spleen and ascites macrophages and ascites fluid (Table 4). In addition, we analyzed cytokine profiles in ascites fluid (Figure 3). M2 macrophages stimulate the production of tumor growth factor β (TGF-β), whereas M1 macrophages are characterized by the production of pro-inflammatory cytokines such as interleukin (IL)-6, IL-12, IL-1β, and TNFα.

Interestingly, the data showed that NO levels were significantly increased in the supernatant of spleen macrophages from TA-treated mice at a dose of 10 mg/kg (*p* < 0.01), as well as in ascites fluid at 5 mg/kg (*p* < 0.01) compared to control animals (Table 4). Conversely, TA at both doses (5 and 10 mg/kg) significantly reduced Arg1 levels in spleen macrophages (*p* < 0.01 and *p* < 0.001, respectively), in ascites macrophages (*p* < 0.05 for both doses), and in ascites fluid (*p* < 0.05 and *p* < 0.01, respectively) (Table 4).

Figure 3 shows cytokine analysis in the ascitic fluid. The increased level of IL-6 was observed in mice treated with TA at a dose of 5 mg/kg (*p* < 0.05), while TA at a dose of 10 mg/kg induced a more pronounced elevation (*p* < 0.01). In contrast, a significantly higher level of TGF-β was detected in the control group compared to TA-treated mice (*p* < 0.001).

### 2.5. TA Exhibits Antiangiogenic Activity

EAT is characterized by high levels of VEGF [1]. VEGF levels were analyzed in ascites macrophages, ascites fluid, and tumor cells (Table 5). Treatment with TA at doses of 5 and 10 mg/kg significantly reduced VEGF levels in ascites macrophages by 83.19% and 74.65%, respectively, compared to the control group. The reduction in VEGF levels in ascites fluid was more modest, 12.32 and 16.75% for the 5 and 10 mg/kg TA groups, respectively. Given the decreased number of tumor cells in the peritoneal cavity following TA treatment and the presence of numerous apoptotic cells and cells with disrupted cellular and nuclear membranes in ascites fluid (see figure), we hypothesized that VEGF levels in tumor cells would be lower compared to ascites fluid. The accumulation of VEGF in ascites may be attributed to its release from damaged or dead tumor cells. Supporting this hypothesis, VEGF levels in tumor cells were reduced by 62.52% and 50.43% following treatment with 5 and 10 mg/kg TA, respectively, compared to the control group.

**Figure 4:**
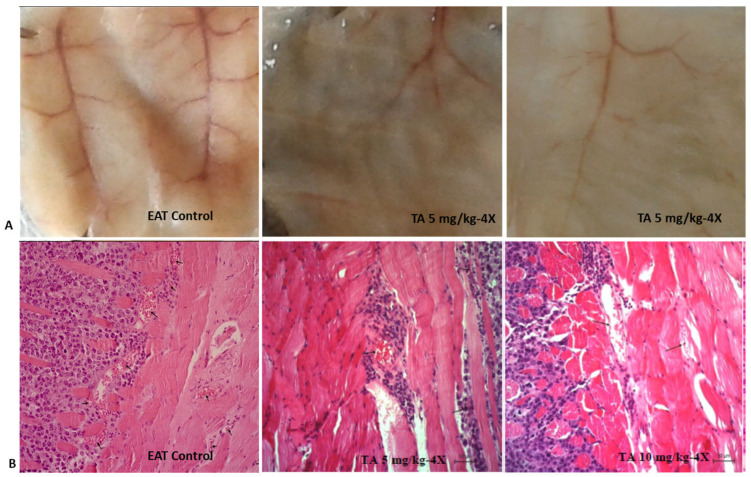
Antiangiogenic effect of tannic acid in vivo. (**A**) Suppression of angiogenesis was clearly observed in TA-treated mice (above). The peritoneal lining of mice treated with saline (0.9%) or TA was inspected for angiogenesis. (**B**) Bouin’s-fixed, paraffin-embedded peritoneal tissue from control and TA-treated mice was sectioned (7 μm), stained with hematoxylin and eosin (H&E), and analyzed for microvessel density (MVD) under a light microscope at ×200 magnification (scale bar = 50 µm). Decreased MVD was observed in the peritoneum of TA-treated mice (below). Abbreviations: EAT, Ehrlich ascites tumor; TA, tannic acid.

High levels of VEGF within the tumor microenvironment promote angiogenesis. As a result, the microvessel density (MVD) in the peritoneal walls of tumor-bearing mice was significantly higher compared to that of healthy mice. Treatment with TA markedly decreased MVD by 78.26% at a dose of 5 mg/kg and by 81.63% at a dose of 10 mg/kg (Table 6, Figure 4).

### 2.6. Tannic Acid Reduces Levels of COX-2 in the Peritoneal Cavity of EAT-Bearing Mice

COX-2 is a key enzyme that promotes angiogenesis and tumor growth. As COX-2 inhibition can block differentiation and polarization of the M2 phenotype [5], we further aimed to determine whether TA inhibits COX-2 expression. To better understand the role of COX-2 inhibition in macrophage function in tumor context, we analyzed levels of COX-2 in ascites macrophages and tumor cells, as well as their interaction with cytokines present in ascites fluid to assess macrophage polarization.

TA treatment at doses of 5 or 10 mg/kg significantly reduced COX-2 levels in ascites macrophages by 85.57% and 78%, respectively (*p* < 0.0001 for both doses), and in tumor cells by 54.56% and 52.17% (*p* < 0.001; *p* < 0.01), respectively (Table 7).

### 2.7. TA Reduces MMP-2 and MMP-9 Levels

MMP-2 and MMP-9 are gelatinases, a specific subgroup of the MMP family that degrade gelatin and collagen, key components of the extracellular matrix (ECM). By degrading ECM components, MMPs facilitate release of VEGF and other factors [2,3,7]. We further investigated whether TA affects the secretion of MMP-2 and MMP-9 using ELISA assay.

The concentrations of both enzymes were markedly lower in ascites macrophages compared to tumor cells. TA treatment at both doses reduced concentrations of MMP-2 in both ascites macrophages and tumor cells, with a more pronounced effect observed in ascites macrophages (*p* < 0.001 for both doses) than in tumor cells (*p* < 0.05 for both doses). On the contrary, although the decreasing trend in MMP-9 expression was observed in ascites macrophages, the effect was not statistically significant. However, expression of MMP-9 was significantly reduced in tumor cells (*p* < 0.05 for both doses) (Table 8).

### 2.8. TA Induces DNA Damage in Tumor and Whole Blood Cells

According to the literature, TA, due to its unique polyphenolic structure, can form complexes with metal ions such as Fe^3+^, Eu^3+^, and Cu^2+^ [28]. Consequently, excessive levels of theses metals in tumor tissue may lead to oxidative stress, DNA damage, and cell death. The alkaline comet assay is a sensitive method used for detection of various forms of DNA damage, as well as for monitoring DNA repair activity and detection of apoptotic or necrotic cells.

Table 9 presents results of the comet assay, showing that TA significantly increased DNA damage in blood cells. TA at both doses (5 or 10 mg/kg) increased the percentage of DNA in the comet tail (*p* < 0.001 for both doses) and tail moment (*p* < 0.001 for both doses) compared to control values. A significant difference in DNA damage was observed between the doses of 5 and 10 mg/kg, possibly due to a higher number of neutrophils at the lower dose.

**Figure 5:**
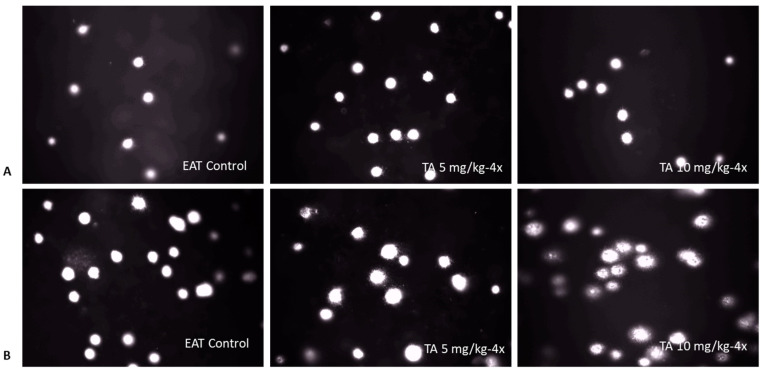
Representative comet assay images showing DNA fragmentation in blood cells (**A**) and Ehrlich ascites tumor (EAT) cells (**B**). Mice were injected intraperitoneally (*ip*) with 2.5 × 10^6^ viable EAT cells and treated with TA at doses of 5 and 10 mg/kg *ip* during exponential tumor growth phase on days 5, 7, 9, and 11. Mice were sacrificed on day 13. A total of 400 cells per group were analyzed and comets were visualized using a fluorescence microscope at 200× magnification.

In tumor cells, TA at a dose of 5 mg/kg increased tail length (*p* < 0.001), percentage of DNA in the tail (*p* < 0.001) and tail moment (*p* < 0.01). Contrary to expectations, TA at a dose of 10 mg/kg did not increase tail length, while for the other two parameters, it induced comparable levels of DNA damage as the 5 mg/kg dose (Table 9). Comet assay representative images of DNA fragmentation in blood and Ehrlich acites tumor (EAT) cells are shown in Figure 5.

### 2.9. TA Effect on Hematological and Biochemical Determinants, Organ Weight and Function

**Figure 6:**
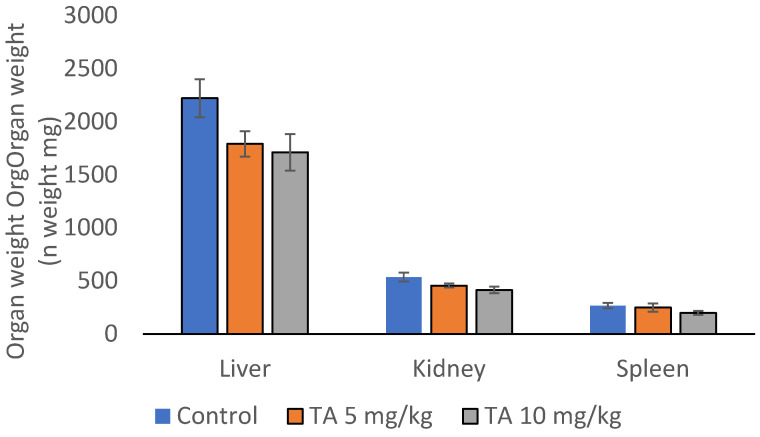
Effect of tannic acid on liver, kidney and spleen weight in mice bearing Ehrlich ascites tumor. Mice were injected intraperitoneally (ip) with 2.5 × 10^6^ viable EAT cells and treated with TA at doses of 5 and 10 mg/kg ip during the exponential tumor growth phase on days 5, 7, 9, and 11. On day 13, mice were sacrificed; liver, kidney and spleen were immediately collected and weighed individually. Data are presented as mean ± SE (*n* = 7 mice per group). Abbreviations: EAT, Ehrlich ascites tumor; TA, tannic acid; SE, standard error.

The biological effects of TA, including its antioxidant, antimicrobial, immunomodulatory and antitumor properties, may exert protective effect on tissues and organs. However, at high doses TA may also exhibit toxic effects. Table 10 and Table 11 show the results of the hematological and biochemical analyses. Treatment of mice with TA did not cause statistically significant changes in any of the hematological or biochemical parameters compared to control (*p* > 0.05). As shown in Figure 6, TA slightly reduced organ weights compared to controls, but the differences were not statistically significant. This probably reflects protective, anti-inflammatory effect of TA against toxic factors released by both cancerous and stromal components of tumor microenvironment.

## 3. Discussion

Malignant ascites, the pathological accumulation of fluid in the peritoneal cavity, is a serious complication of advanced-stage cancers. It is associated with poor prognosis, the survival following diagnosis is typically up to 20 weeks, together with the prominent impairment of quality of life. Malignant ascites is often sign of peritoneal carcinomatosis, where cancer cells spread across the lining of abdominal cavity. EAT, a fast-growing tumor model, exhibits characteristics such as extensive neoangiogenesis, local inflammation, high VEGF levels and increased vascular permeability. These characteristics closely resemble those of human breast tumor and advanced-stage epithelial ovarian cancer, but also peritoneal tumors including colon, pancreas and uterus; tumors of extra-abdominal origin such as lymphoma and lung cancer; and a small number of cancers of unknown primary origin.

Biological activities of TA are largely attributed to the presence of numerous hydroxyl groups and aromatic benzene rings, enabling various interactions, including hydrogen bonding, electrostatic, coordinative, and hydrophobic interactions. The hydroxyl groups in hydrolyzable tannins HTs/TA can act as both hydrogen donors and acceptors [29], supporting multiple molecular interactions. The ability of TA to bind to intracellular target proteins and DNA makes it a potential agent in enzyme inhibition, chemoprevention, chemosensitization, drug delivery, and modulation of signaling pathways. It has been shown that TA interferes with several critical pathways involved in cancer development and progression, including NF-κB, PI3K/AKT, ERK1/2, Wnt/β-catenin, JAK/STAT, RAS/RAF/mTOR, TGF-β1/TGF-β1R axis, VEGF/VEGFR signaling and CXCL12/CXCR4 axis [16,30,31,32,33,34]. Dysregulation of these pathways in tumor cells leads to uncontrolled cell growth, increased proliferation, inflammation, inhibition of apoptosis, altered metabolism, increased survival, angiogenic effect, metastasis and drug resistance. TA effectively inhibits Ras/Raf/MEK/ERK and Ras/PI3K/PTEN/Akt/mTOR signaling pathways, which are crucial in the transmission of proliferative signals from membrane-bound receptors. Furthermore, TA is a negative regulator of signaling pathways associated with cancer-cell metastasis and therapy resistance, including the TGF-β, Notch, Wnt, AKT-mTOR, MAPK/ERK, and NF-κB pathways [32,33]. The anti-angiogenic and antimetastatic properties of TA are further supported by selective inhibition of VEGF/VEGF signaling and antagonistic effects on CXCL12/CXCR4. By inhibiting the Jak2/STAT3 pathway, TA induces G1/S cell-cycle arrest and initiates the caspase-dependent mitochondrial apoptotic pathway [16]. In addition, many effects of the Ras/Raf/MEK/ERK and Ras/PI3K/Akt/mTOR pathways on apoptosis are mediated by ERK or Akt phosphorylation of key apoptotic effector molecules (e.g., Bcl-2, Mcl-1, Bad, Bim, CREB, Foxo, Caspase-9, among others). TA treatment significantly reduces the phosphorylation of ERK1/2, AKT, and p38, leading to decreased viability of cancer cells and increased percentage of apoptotic cells. This is accompanied by decreased expression of antiapoptotic proteins (Bcl-2, Mcl-1, and Bcl-XL), resulting in the caspase-3 activation [34]. Although the anticancer effects of TA have been confirmed in various tumor models [19,20,21,22,23,24,35,36,37,38], data on its efficacy in malignant ascites are still limited.

Based on this, the study aimed to provide a deeper insight into the molecular processes underlying effects of TA on tumor growth, angiogenesis and immunomodulation, with an emphasis on macrophage polarization, levels of VEGF, MMP-2, MMP-9 and COX-2, microvessel density, and cytokine release, as well as its potential to induce DNA damage in tumor and blood cells.

Our results demonstrate that TA, at doses of 5 or 10 mg/kg, exhibits an antitumor effect: (i) TA significantly suppressed proliferation of EAT cells, reducing tumor growth by approximately 75%, (ii) TA reduced VEGF levels in ascites macrophages by 83.19% and 74.65% and in tumor cells by 62.52% and 50.43%, (iii) TA decreased MVD by 78.26% and 81.63%, (iv) TA inhibited COX-2 expression in ascites macrophages by 85.57% and 78%, and in tumor cells by 54.56% and 52.17; (v) TA reduced MMP-2 levels by 71.35% and 84.22%, as well as MMP-9 levels by 69.15% and 48.64% (Table 1, Table 2, Table 3, Table 4, Table 5, Table 6, Table 7, Table 8 and Table 9, Figure 1, Figure 2, Figure 3 and Figure 4).

Interestingly, although the number of cells in the abdominal cavity was reduced, the volume of ascites fluid remained unchanged (Table 1, Figure 1). This may be attributed to the fixative properties of TA, and the advanced stage of tumor progression at which mice were analyzed (5, 7, 9, 11 days post-inoculation). It is well-documented that mice typically begin to die around day 9 post-inoculation. When injected intraperitoneally, the EAT progresses aggressively, resulting in rapid mice mortality, much faster compared to subcutaneous or intramuscular inoculation. The lethality of this tumor model is related to rapid proliferation, impaired apoptosis, increased angiogenesis and immunosuppression, and widespread invasion and metastasis, all of which contribute to mortality in human cancers [7]. TA appears to induce apoptosis and necrosis, interfere with essential cellular processes (Figure 2), and trigger oxidative stress and DNA damage in tumor cells (Table 9). Differential cell counts revealed a marked reduction in tumor cells in peritoneal cavity (Table 1 and Table 2), suggesting that apoptosis and necrosis could be the key mechanism underlying the antitumor effect of TA, as supported by previous studies [11,12,13,14,15,16,37,38].

Moreover, TA significantly suppressed the process of angiogenesis (Table 5 and Table 6, Figure 4), an early event that is critical for tumor development. Angiogenesis is driven by high levels of VEGF produced by both tumor cells and cells of the tumor microenvironment, including TAMs and tumor-associated neutrophils (TANs). TAMs and TANs contribute to cancer pathogenesis by releasing MMP-9, VEGF and other specific pro-tumorigenic factors (e.g., neutrophil elastase), which further promote cancer cell invasion, angiogenesis, and metastasis. There is also a complex regulatory relationship between VEGF and MMPs. Some studies suggest that interplay between VEGF and MMP-2 contributes to ascites accumulation, while others highlight the role of VEGF and MMP-9 in this process. Specifically, VEGF upregulates MMP-9 expression, while VEGF inhibition can suppress MMP-9 activity, ascites volume and tumor burden [2,5,7].

Hypoxia, an important characteristic of the ascites tumor microenvironment, directly stimulates VEGF expression via hypoxia inducible factor (HIF), while indirectly inhibits the maturation and function of dendritic cells (DC) and macrophages. VEGF acts in both autocrine and paracrine manner as a potent mitogen that supports proliferation of EAT cells in vivo. Angiogenesis plays a pivotal role in tumor growth and metastatic spread. As shown in Table 6 and Figure 4, the MVD in the peritoneal walls of tumor-bearing mice was significantly higher than in TA-treated mice. TA at both doses (5 or 10 mg/kg) effectively reduced VEGF levels and blood vessel proliferation in the peritoneal wall, as well as MMP-2/-9 expression (Table 8). These results are consistent with the TA-mediated inhibition of MMP-2/-9 observed by zymography assay [39]. Previous research has also reported that phenolic compounds, including TA, may inhibit the proteolytic activity of MMP-2 and MMP-9 [2,40,41]. Chiang et al. [42] elucidated the molecular basis of interactions between TA and MMPs; they are based on the formation of H-bonds and hydrophobic and electrostatic interactions. Specifically, TA interacts with MMP-2 by forming hydrogen bonds with two digallic acid arms, and with MMP-9 via four digallic acid arms, resulting in more stable non-covalent interactions in the TA-MMP-9 complex compared to the TA-MMP-2 complex.

Macrophages, as key components of the tumor microenvironment, interact with tumor cells through a variety of signaling molecules, including growth factors, cytokines, and proteolytic enzymes such as MMPs. They can polarize toward either the classically activated M1 phenotype with tumor-suppressive properties, or the alternative M2 phenotype that promotes tumor progression [2,3,7,41]. TAMs predominantly display features of M2 macrophages. They stimulate tumor progression by enhancing the angiogenesis process via VEGF, recruit macrophages via macrophage colony-stimulating factor (M-CSF) or TGF-β, and promote invasion via MMPs [2,3,7,41]. Therefore, therapeutic strategies capable of reprogramming their polarization towards the M1 phenotype have been recognized as potential approach in overcoming resistance to chemo- and radiotherapy [43].

Our results suggest that the TA-induced decrease in VEGF secretion in tumor cells and ascites macrophages likely contributes to reduced blood vessel formation (Table 5 and Table 6). Furthermore, previous studies have shown that COX-2 inhibition reduces vascular density [2]. As COX-2 plays a critical role in M2 polarization, tumor growth and angiogenesis through the VEGF upregulation [44,45], these findings suggest a mechanistic explanation of antitumor TA activity.

Numerous studies [2,4,5,44,45] have shown that COX-2 may facilitate tumor progression by stimulating cancer cell proliferation, inhibiting apoptosis of cancer cells, enhancing invasion and angiogenesis, and suppressing immune responses, particularly when expressed by TAMs. Macrophages, especially those polarized to the M2 phenotype, create a tumor-promoting microenvironment, while COX-2 influences this polarization and enhances the pro-tumoral effects of M2 macrophages, suppressing the anti-tumor immune response. Our findings indicate that TA stimulates the immune response, increases the spreading index of macrophages, upregulates NO levels while decreasing Arg1 activity, and reduces amounts of COX-2, MMP-2 and MMP-9, altogether indicating a shift from the M2 polarization (Table 4, Table 7 and Table 8). Our observations in a murine model are consistent with the findings of Na et al. [5], who demonstrated that COX-2 inhibition results in the loss of the M2 characteristics in TAMs and may contribute to the prevention of breast cancer metastasis [5]. It has been demonstrated that VEGF-A and VEGF-C can be induced by COX-2. These data indicate that COX-2 inhibition suppresses lymph node metastasis by reducing macrophage mediated lymphangiogenesis [46]. In addition, the inhibition of COX-2 reduces the production of pro-inflammatory mediators, which is also evident in our results (Figure 3).

We observed an increase in IL-6 levels. IL-6 has a dual role in the tumor microenvironment. On one hand, it is recognized as a key player in the activation, proliferation and survival of lymphocytes during active immune responses. Fisher et al. [47] reported that IL-6 promotes T cell trafficking to lymph nodes and tumor site, enhancing cytotoxic effector functions. IL-6 regulates the activity of immune cells by promoting differentiation of naïve CD4+ T cells into Th17 cells and B cells into antibody-producing plasma cells, and enhancing the cytotoxic activity of CD8+ T cells [18,48,49,50,51]. Furthermore, IL-6 increases the expression of adhesion molecules, such as ICAM-1, VCAM-1 and E-selectin on endothelial cells, as well as L-selectin on lymphocytes, which results in increased transmigration of leukocytes into the tumor area [48,49]. The increase in antibody production stimulates antibody-dependent cell-mediated cytotoxicity (ADCC), activating NK cells and macrophages to recognize and destroy cancer cells [18,49,50]. In addition to NK cells and monocytes/macrophages, other effector cells such as NKT cells and γδ T cells can also contribute to this process [18,50,51]. Th-17 cells may further enhance anti-tumor immunity by recruiting additional immune cells to the tumor site [48]. On the other hand, IL-6 can also participate in immune evasion by upregulating PD-L1, potentially compromising drug efficacy [47]. Given these opposing effects, further research is needed to clarify the role of IL-6 in immune modulation.

According to our data, TA treatment at a dose of 10 mg/kg significantly increases the number of lymphocytes, apparently stimulating cytotoxic immune responses (Table 2 and Table 3). LMR is considered a useful marker of the immune host response against cancer. A higher LMR generally indicates better overall prognosis and survival, while a lower LMR is an indicator of a week immune response and increased number of monocytes, which often correlate with a tumor-promoting microenvironment and tumor burden [52,53]. Tumor microenvironment can modulate immune cell behavior, including neutrophil recruitment and polarization. TANs are known to increase tumor growth, invasiveness and metastasis in various solid tumors. They are important contributors to cancer pathogenesis, and important for establishing an immunosuppressive environment. They promote tumor growth and metastatic capacity, often through the formation of neutrophil extracellular traps [54]. In addition, TANs are a major source of MMP-9, which degrades the extracellular matrix and releases growth factors that promote angiogenesis. High NLR is often observed in malignant conditions, including malignant ascites, and serves as a poor prognostic factor. High NLR is linked to systemic inflammation, which can drive cancer progression by promoting cell proliferation, metastasis, treatment resistance, and increased risk of recurrence across various cancers. Our data demonstrated that NLR in the control group was almost three times higher compared to TA-treated mice (5 and 10 mg/kg), suggesting that TA may attenuate systemic inflammation. This finding is consistent with the observed immunomodulatory and anti-inflammatory properties of TA, supporting its potential role as an adjuvant therapy against cancer.

As previously mentioned, TA possesses antioxidant activity and may interfere with various metabolic pathways in tumor cells. TA can also act as pro-oxidant under certain conditions, resulting in oxidative DNA damage. Polyphenols, including TA, might become sources of ROS through the formation of semiquinone and quinone oxidative products, depending on the extra- and intracellular environment, particularly pH and the presence of metal ions [11,12,13,16,41]. Our results showed that TA induces DNA damage in tumor cells and blood cells, probably by increasing ROS levels (Table 9). Importantly, the extent of DNA damage was higher in tumor cells than in blood cells. To assess DNA damage, we used the comet assay, also known as single-cell gel electrophoresis, under alkaline conditions. By measuring cytotoxic or genotoxic effects using the comet assay, it is possible to determine the effectiveness of therapy in cancer cells, while in healthy cells, the comet assay serves as a standard method for assessing potential harmful effects [55,56]. It is important to distinguish between cytotoxic effects, which affect cell viability and may involve apoptosis, and genotoxic effects, which refer to damage to DNA and other cellular elements that maintain genome stability. The cytotoxic or genotoxic potential often depends on the dose or concentration used. To differentiate between cytotoxicity and genotoxicity using the comet test, the relative change (fold change, FC) of specific parameters is used, most commonly the tail moment. The tail moment, defined as the product of tail length and the percentage of DNA in the tail, provides a comprehensive measure of DNA damage [56,57]. According to Daza et al. [57], the mean tail moment values of cytotoxic substances were 0.8–1.07, while genotoxic substances were characterized by significantly higher tail moments. For example, for actinomycin C, tail moment ranged from 5.16 to 8.27, and for camptothecin from 6.30 to 12.13, depending on the applied concentration. In our study, we determined that in both TA-treated groups, the obtained tail moment values fall within the range of cytotoxic effects, excluding a genotoxic effect at the tested concentrations. These findings suggest that TA-induced DNA damage is likely a secondary consequence of cytotoxicity (via ROS-induced apoptosis or necrosis) rather than a result of direct genotoxic interaction with DNA.

The effects of TA on whole blood cells as well as tumor cells can be attributed to its unique structural characteristics, which enable electrostatic interactions, hydrogen bonding and hydrophobic interactions with various biomolecules. Its metal-chelating properties, primarily via the catechol or galloyl groups [28], also play an important role. For example, TA can form coordination complexes with at least 18 metal ions, such as Fe^3+^, Eu^3+^, and Cu^2+^. Tumor cells are known to accumulate iron, which is essential for their proliferation and survival. Excess iron in cancer cells can catalyze the Fenton reaction, generating highly reactive hydroxyl radicals (OH), which contribute to oxidative stress and DNA damage. This oxidative environment can promote chronic inflammation and signaling pathways that support cancer development and progression. It can also lead to lipid peroxidation and membrane damage, ultimately triggering ferroptosis, a form of regulated cell death. Similarly, copper levels are also elevated in serum and tumor tissue (~20%) of cancer patients compared to healthy individuals. Copper levels are closely associated with tumor growth, angiogenesis and metastasis. Maintaining copper redox homeostasis is critical because disruption can enhance the production of free radicals, leading to damage of lipids, proteins, DNA, and other biomolecules, which ultimately results in cell damage. Disruption of copper metabolism in cancer can affect mitochondrial respiration, glycolysis, insulin signaling, and lipid metabolism. Excess copper levels, through the redox cycling between Cu^+^/Cu^2+^ and Fe^2+^/Fe^3+^, can initiate Fenton-like reaction and production of hydroxyl radicals, leading to oxidative damage and cell death via cuproptosis or ferroptosis [58].

In line with these findings, it has been shown that TA interferes with lipid signaling and metabolism of prostate cancer cells. Specifically, TA suppressed lipid metabolic pathways, induced ROS accumulation and endoplasmic reticulum (ER) stress, and ultimately triggered apoptosis in these cells [59]. Erythrocytes are particularly sensitive to oxidative stress due to their high content of unsaturated fatty acids, which are prone to lipid peroxidation. In addition, they are endogenous source of ROS, mainly produced because of NADPH oxidase activation and hemoglobin autooxidation. It is possible that TA interacts with Fe released during erythrocyte hemolysis, leading to generation of hydroxyl radicals via Fenton chemistry. This may contribute to oxidative DNA damage in whole blood cells.

According to our results, TA can inhibit numerous molecular targets associated with tumor growth and angiogenesis, including cytokines, COX-2, MMP-2/MMP-9, and VEGF. Given the multiple mechanisms of its action in the inhibition of tumor growth and angiogenesis, TA deserves further investigation, particularly regarding the interaction between HIF/VEGF and immune cell, and its capacity to form semiquinone and quinone oxidative products depending on pH and the presence of oxidants, including the metals with variable oxidation states. The possibility of easy formation of radicals in tumor cells may give TA an advantage as a potential candidate for pro-oxidant cancer therapy through the induction of ROS-mediated cell death.

Despite the good characteristics of the EAT model for the assessment of ascites tumors, there are a number of limitations. The main limitation of this model is the absence of tumor-specific transplantation antigen (TSTA), which prevents the development of a complex immune response in the host [25,60,61,62]. Other limitations include the rapid growth and aggressiveness of tumors, reduced heterogeneity, inability to metastasize, and lower genetic diversity both within a single tumor and between different tumors compared to human cancers. The model is not suitable for studying slow-growing tumors or evaluating long-term toxicity. In contrast, human cancers are characterized by high heterogeneity, diverse genetic mutations, the ability to metastasize, complex immune response and variable tumor growth rates [25,60,61,62]. The results obtained should be further investigated and validated in more suitable and complex models that better reflect the complexity of human tumors. Patient-Derived Xenografts model (PDX) models in immunodeficient mice could serve as a valuable model for translating preclinical findings into clinical application in ascites cancer in humans. However, they lack the complex interactions between cancer cells and their surrounding environment. Furthermore, genetic, histological, and microenvironmental differences between xenografts and human tumors may lead to differences in treatment response [63]. Nevertheless, the PDX model could be suitable for preclinical investigation of the dose-dependent effect and possible toxicity of TA during long-term administration, considering that high doses can be toxic [64]. Daily intake of tannins below the range of 1.5–2.5 g is considered safe and generally does not cause any adverse effects in humans, while consumption above this range may reduce the bioavailability of nutrients in the intestine and impair the absorption of iron or other essential metals from the diet [64]. Our data demonstrate that TA administered at doses of 5 and 10 mg/kg in mice did not induce changes in hematological and biochemical parameters (Table 10 and Table 11), nor did it affect liver, kidney and spleen weights or their functions (Figure 6). These findings support the protective and anti-inflammatory effects of TA against toxic factors released by both carcinogenic and stromal components of the tumor microenvironment.

Future research should also focus on macrophage polarization in human peripheral blood mononuclear cells (PBMCs), assess receptor and cytokine expression in human and murine macrophages using flow cytometry, and investigate potential mechanisms of TA on immune cells within the tumor microenvironment. Furthermore, analysis of different forms of cell death, including apoptosis, necrosis, autophagy, and specific types such as cuproptosis or ferroptosis, would add valuable insights into the antitumor efficacy of TA. However, it should be noted that that the inhibition of tumor growth, together with the suppression of MMPs, COX-2, M2 polarization, VEGF, and the number of blood vessels, combined with the enhancement of the immunopotentiating blood biomarkers such as lymphocyte-to-monocyte ratio (LMR) and the reduction in the polymorphonuclear/mononuclear (P/M) ratio, provides a strong basis for further preclinical research.

In addition to its potent chemopreventive and anticancer properties, TA has great potential in chemosensitization to overcome multidrug resistance (MDR) and reduce chemotherapy-associated toxicity through multiple mechanisms, including anti-inflammatory and antioxidant effects. TA can counteract MDR in chemotherapy by reducing the activity of efflux pumps such as P-gp, MRP1 and MRP2, thereby inhibiting cellular efflux pathways [20,29,38]. The inhibition of proteasome activity by TA provides an additional mechanism for overcoming drug resistance, further supporting its potential in chemotherapy.

Furthermore, the unique structure of TA makes it a suitable candidate for use as a crosslinker in the design of various anticancer nanoparticles and hydrogels. The use of TA/Fe^3+^ complexes as a coating agent for the preparation of nanoparticles gives them additional functions such as photothermal conversion, and chemodynamic and theranostic capabilities. Therefore, TA has been used in the preparation of cross-linked hydrogels and TA-based nanoparticles including liposomes, bioceramics, polymers, micelles, carbon nanotubes, mesoporous silica, etc. Acting as a hydrogen bond (HB) donor, TA can interact with synthetic and natural polymers to form multilayer films, microcapsules or microparticles through hydrogen bonding [29,38].

Future studies should be directed toward elucidating the structural properties and molecular interactions of TA in order to improve biological functionality, especially interactions between TA and metal nanostructures, as well as TA and polymers, their efficacy, synergism with other therapies, and possible toxicity to enable broader clinical application. In addition, hydrophobic interactions have been detected in the assembly of hydrolyzable tannins (HTs) with proteins such as lysozyme and glucose oxidase (enzymes with catalytic activity), cytochrome C (a transport protein), and immunoglobulin G (an antibody). These interactions can alter protein structure, thereby influencing stability, digestibility, and functionality. By optimizing parameters such as molecular weight, hydrophobicity, and electrical charge, TA-based materials can be customized for a variety of biomedical applications [29].

## 4. Materials and Methods

### 4.1. Experimental Animals and Ethics Statement

The study used male Swiss albino inbred mice, obtained at the Department of Animal Physiology, Faculty of Science, University of Zagreb. The mice, weighing 20–25 g and approximately 2 months old, were kept in cages with a maximum of 7 animals under standard laboratory conditions (12 h light/12 h dark cycle, temperature maintained at 24 °C with controlled humidity). Animals had constant access to standard rodent chow (Standard Diet GLP, 4RF21, Mucedola, Settimo Milanese, MI, Italy) and water. The study and all animal procedures were reviewed and approved by the Ethical Committee of Faculty of Science, University of Zagreb (approval code: 251-58-10617-16-1; date of approval: 17 December 2014). The research was conducted in accordance with the ethical and legal standards of the European Union [65,66] and the Republic of Croatia [67,68,69,70] and according to the Guide for the Care and Use of Laboratory Animals, DHHS (NIH) Publ # 86–23, National Research Council) [71].

### 4.2. Tumor Cells

Ehrlich ascites tumor (EAT) cells were maintained in vivo by serial intraperitoneal (*ip*) transplantation in Swiss albino mice every 7 or 9 days, forming ascites tumor. To collect the cells, the abdominal cavity was rinsed with 3 mL of saline, followed by gentle massage of the abdominal wall. An incision was then made, and the peritoneal fluid was collected using a Pasteur pipette. Cells obtained from the peritoneal fluid were diluted with saline (0.9% sodium chloride solution) to obtain a final concentration of 2.5 × 10^6^ EAT cells/0.5 mL. The number of viable cells was determined using trypan blue and a Bürker–Türk chamber. Cell viability was calculated using the following equation:

$$\mathrm{Viability}\mathrm{of}\mathrm{EAT}\mathrm{cells}\;(\%)\;=\;\frac{No\;of\;viable\;cells}{Total\;No\;of\;viable\;and\;dead\;cells}\;\times 100$$

A cell viability threshold of 95% or higher was considered acceptable for further experiments.

### 4.3. Tannic Acid

**Figure 7:**
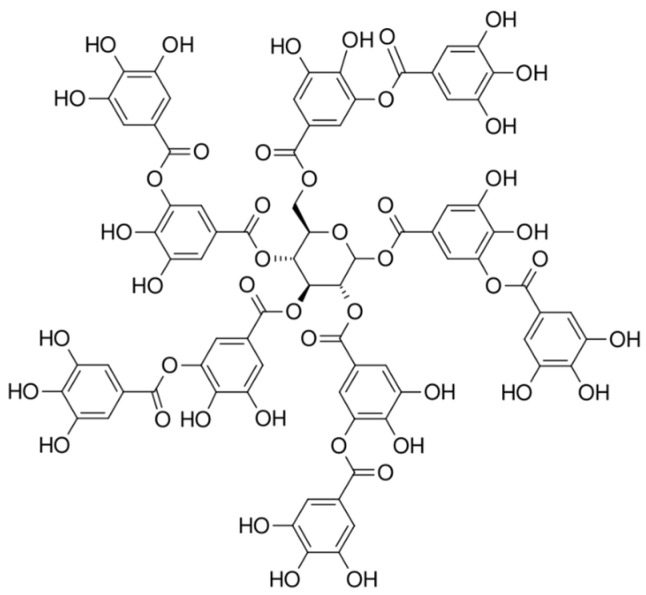
Tannic acid structure.

Tannic acid (C_76_H_52_O_46_, Mr = 1701.19, purity > 97%) manufactured by Merck KGaA, Darmstadt, Germany, was dissolved in saline (B. Braun Adria d. o. o., Zagreb, Croatia) and injected into mice at doses of 5 mg/kg and 10 mg/kg. Figure 7 shows the chemical structure of tannic acid.

### 4.4. Experimental Design and Animal Treatment

After initial weighing, the animals were randomly divided into three groups (n = 7 per group) and injected *ip* with 2.5 × 10^6^ EAT cells on day 0 of the experiment. Treatment of EAT-bearing mice started on day 5, when the progression of EAT cell growth became apparent. Groups 2 and 3 received TA at doses of 5 and 10 mg/kg *ip*, on days 5, 7, 9 and 11. Group 1 served as the control and was treated *ip* with saline. Animals were sacrificed on day 13, and samples of blood (serum), ascites fluid, and target organs (kidney, liver, spleen, and peritoneal membrane) were collected. During this procedure, all animals were adequately anesthetized and analgesed via *ip* injection of a combination of Narketan^®^ (Vetoquinol S.A. BP 189, Lure Cedex, France) and Xylapan^®^ (Vetoquinol Biowet Sp., Gorzow, Poland) at a dose of 100 mg/kg and 5 mg/kg, respectively. Before sacrifice, the body weight was measured. Following sacrifice, the volume of ascites fluid collected from the abdominal cavity was used for the analysis of the total cell number in the abdominal cavity, the volume of ascitic fluid, expression of VEGF, MMP-2, MMP-9, COX-2, NO and Arg1, and cytokine concentrations. Kidneys, liver, and spleen were collected and weighed for protective effect analysis, while spleen was used for macrophage analysis. A part of the peritoneal wall was excised, fixed, and used for histological analysis and evaluation of MVD.

### 4.5. Monitoring Changes in Body and Organs Weight

The animals were weighed on days 0, 5, and 13 using digital scale. Weight changes were monitored to assess weight loss or gain compared to the control group. Percentage change in body weight was calculated for each animal using the following formula:

$$Percentage\;change\;in\;weight\;=\;\frac{\left(Final\;weight\;-\;Initial\;weight\right)}{Final\;weight}\;\times 100$$

Kidneys, liver, and spleen were collected and individually weighed to assess the possible toxic, inflammatory/anti-inflammatory effect of TA.

### 4.6. Antitumor Efficacy of TA

#### 4.6.1. Determination of the Total Volume of Peritoneal Fluid, Tumor Cell Counts and Tumor Inhibition

The volume of ascites fluid and the number of cells in the abdominal cavity were analyzed 13 days after the injection of EAT cells. An incision was made in the abdominal region of each animal, and the peritoneal fluid was individually collected. The volume of the fluid and total number of cells were determined using a Bürker–Türk counting chamber. Tumor growth inhibition was calculated using the following formula:

$$Tumor\;inhibition\;\left(\%\right)=\frac{\left(Av.no.of\;cells\;in\;control\;group-Av.no.of\;cells\;in\;exp.group\right)}{Average\;number\;of\;cells\;in\;control\;group}\;\times 100$$

#### 4.6.2. Differential Cell Analysis in Peritoneal Fluid

Cell smears from the peritoneal cavity were air-dried at room temperature and stained using May-Grünwald and Giemsa solutions (1 part Giemsa: 2 parts distilled water). The preparations were analyzed under a light microscope at 400× magnification. Differential cell analysis was performed by counting lymphocytes, macrophages, neutrophils, eosinophils and basophils, calculated per 100 cells. At least 800 cells per group were analyzed.

#### 4.6.3. Analysis of Hematological and Biochemical Parameters

Blood samples were collected from the axillary vein into EDTA-containing vacutainers. Red blood cells (RBC), hematocrit (HCT), hemoglobin (HB), mean cell volume (MCV), mean cell hemoglobin (MCH), and mean cell hemoglobin concentration (MCHC), erythrocyte size distribution (RDW), platelets (Plt), mean platelet volume (MPV), white blood cells (WBC) and differential blood analysis were determined using a Abbott Cell Dyn 3700 electronic counter (Abbott Diagnostics, Chicago, IL, USA). All hematological parameters were analyzed, but only the total number of white blood cells (WBC count) and differential blood analysis are shown here. The neutrophil-to-lymphocyte (NLR) and lymphocyte-to-monocyte (LMR) ratios were calculated from absolute counts. Polymorphonuclear/mononuclear (P/M) ratio was calculated based on the total number of polymorphonuclear and mononuclear cells.

For biochemical analysis, additional blood samples parts were collected and immediately placed on ice. Serum was obtained by centrifugation at 2200 rpm for 10 min. It was used for the estimation of liver enzyme activity and kidney function. Biochemical parameters included glucose, urea, total bilirubin (TBIL), C-reactive protein (CRP), and the activities of aspartate aminotransferase (AST), alanine aminotransferase (ALT), lactate dehydrogenase (LDH) and amylase (AMY). Serum biochemical parameters were determined on a Beckman Coulter AU 680 biochemical analyzer (Beckman Coulter, Brea, CA, USA).

### 4.7. Effect of TA on Angiogenesis

#### 4.7.1. Histological Analysis of the Peritoneum and Micro Vessel Density (MVD)

After sacrificing the mice, cells were isolated from the peritoneal cavity, and a part of the peritoneum was excised, washed in saline, and fixed in Bouin’s fixative (75 mL saturated picric acid, 25 mL 40% formaldehyde, 5 mL glacial acetic acid) for 24 h. After fixation, peritoneal samples were thoroughly washed in tap water and dehydrated by immersion in increasing concentrations of ethanol (50%, 70%, 80%, 90%, 95%, 100%), with the addition of a few drops of ammonia solution. Clarification was performed by placing the samples in chloroform for 24 h. Peritoneal samples were then embedded in paraplast and cut using a microtome into 7–8 μm thick sections. Sections were placed onto microscope slides using a heated water bath (40 °C), then deparaffinized by immersion in xylene (2 times for 15 min), rehydrated by immersion into decreasing concentrations of ethanol (100%, 96%, 80%, 70% for 5 min each), and finally rinsed with distilled water (twice for 5 min). Slides were stained with Mayer’s hematoxylin (hemalaun) solution for 8 min followed by 0.5% eosin for 3–4 min according to a standard protocol [2]. After staining, slides were dehydrated through increasing ethanol concentrations (70%, 80%, 96%, 100%) and xylene, embedded in Canada balsam, and allowed to dry for 24 h. Stained slide sections from comparable areas were analyzed under a Nikon Eclipse E600 light microscope at 40, 100, and 200× magnification.

#### 4.7.2. Quantitative Measurement of Vascular Density

Quantitative measurement of peritoneal vascularity was performed by calculating the average microvascular density (MVD) in the areas of most intensive vascularization. According to Weidner’s recommendations, MVD was determined in areas of invasive tumor containing the highest number of capillaries and microvessels per unit area. The areas with the highest blood vessel density (“hot spots”) were analyzed under a minimum magnification of 10×, and individual microvessel counts were performed at 400× magnification. The results were expressed as the highest number of microvessels observed within a single 200× field. Photomicrographs were taken using a digital camera AxioCam ERc5s, Zeiss (Carl Zeiss Microscopy GmbH, Göttingen, Germany) and processed with ZEISS ZEN 2 morphometric image analysis software [72]. The results included intact blood vessels as well as endothelial cell bands and individual endothelial cells. Microvessel counts were performed independently by two investigators.

### 4.8. Isolation and Preparation of Macrophages from Ascites Fluid and Spleen

Ascites fluid from treated and control animals (1 × 10^6^ cells/mL) was incubated for 3 h at 37 °C with 5% CO_2_ in 24-well plates. Following incubation, the supernatant containing tumor cells was removed. Active macrophages, adhered to the bottom of the wells, were gently washed with saline and further incubated in 1 mL of DMEM medium supplemented with 10% FBS for 24 h under the same conditions. After 24 h, the supernatant was collected and stored at −20 °C for the analysis of NO, Arg 1, COX-2, MMP-2, MMP-9 and VEGF. Tumor cells were used for VEGF, COX-2, MMP-2, and MMP-9 analyses.

Spleens were excised from the abdominal cavity, weighed, and processed into a single-cell suspension. The mononuclear cells were extracted using Lymphoprep (Nyegard, Oslo, Norway). The mononuclear cell layer was collected, washed twice with medium and adjusted to 1 × 10^6^ cells/mL with 0.1 g/mL LPS in DMEM containing 10% FCS, and incubated at 37 °C and 5% CO_2_ for 24 h. The supernatant was then stored at −20 °C for subsequent NO and Arg1 analysis.

The preparation protocols for tumor cells, peritoneal macrophages, and spleen macrophages were based on methfods described by Oršolić et al. [2]. Briefly, adherent macrophages were detached using 0.025% trypsin and incubated for 3 min. Trypsin activity was neutralized by adding 1 mL of DMED medium with 10% FBS. Cells were then detached using a cell scraper and stored at −20 °C for further analysis.

#### 4.8.1. Determination of Functional Activity of Peritoneal Macrophages

To analyze macrophage functional activity, peritoneal fluid smears were prepared on slides (4 slides per experimental group). After air-drying at room temperature for 15–20 min, the slides were washed with PBS to remove non-adherent cells. The remaining adherent cells were incubated for 1 h in DMEM medium (Gibco; Thermo Fisher Scientific, Inc., Waltham, MA, USA) containing 10 nM HEPES buffer at 37 °C. Following incubation, the medium was removed, and the cells were fixed with 2.5% glutaraldehyde. The slides were then stained with 5% Giemsa solution for 15 min. The stained slides were analyzed under a light microscope, and the proportion of activated macrophages relative to non-activated macrophages was determined using the spreading technique [2]. A total of 200 macrophages were evaluated and classified as either round or spread. The macrophage spreading (SI) index was calculated as follows:

*SI* = (*number of spreading macrophages*/200 *adherent cells*) × 100

#### 4.8.2. Nitric Oxide Analyses by Griess Reaction

In M1 macrophages, NO is produced by inducible nitric oxide (iNOS). NO levels were measured using the Griess reaction that determines concentrations of nitrate and nitrite produced, employing a commercial nitrate/nitrite assay kit (Griess Reagent System, Promega, Madison, WI, USA). The Griess reaction is based on a chemical reaction between sulfanilamide and N-1-naphthylethylenediamine dihydrochloride in an acidic phosphoric acid environment, resulting in the formation of azo-colored compounds. The absorbance was recorded at 540 nm using a microplate spectrophotometer (Ao Absorbance Microplate Reader, Azure Biosystems, Dublin, CA, USA). A sodium nitrite (0.1 M) standard curve was generated using concentrations ranging from 0 to 100 μM. The absorbance values of the samples were averaged and converted to nitrite concentrations (µM) based on the standard curve of sodium nitrite. The assay’s limit of detection was 2.5 μM.

#### 4.8.3. Arginase Activity (Arg)

Arg 1 is considered a classical anti-inflammatory marker in M2 macrophages and is involved in attenuating inflammatory responses of macrophages. Arginase activity (Arg) was measured in cell supernatants using a commercial Arginase activity assay kit MAK112 (Sigma-Aldrich, St. Louis, MO, USA), as previously described [2,37] and following the manufacturer’s instructions. In this assay, the arginase catalyzes the conversion of L-arginine into urea and ornithine. One unit of arginase activity is defined as the amount of enzyme that converts 1.0 μmole of L-arginine to ornithine and urea per min at 37 °C and pH 9.5. Upon the addition of the urea reagent, a colored product is formed, proportional to arginase activity. Urea concentration was determined spectrophotometrically by measuring absorbance at 430 nm (Ao Absorbance Microplate Reader, Azure Biosystems, USA).

### 4.9. Determination of Proangiogenic Factors: VEGF, MMP-2, MMP-9 and COX-2

#### 4.9.1. VEGF Analysis

VEGF levels were determined in ascitic fluid, tumor cells, and ascitic macrophages using the Mouse VEGF Quantikine^®^ ELISA kit (R&D Systems Europe, Ltd., London, UK) according to the manufacturer’s instructions. Briefly, ascitic fluid and tumor cell samples were diluted 2-fold using the Calibrator Diluent RD5T (R&D Systems Europe, Ltd., London, UK) provided in the kit, while ascitic macrophage cells were used undiluted. Assay Diluent RD1N buffer (R&D Systems Europe, Ltd., London, UK) (50 μL) and 50 μL of standard or appropriate sample were added to a 96-well microtiter plate and incubated for 2 h at room temperature to allow binding of VEGF to the immobilized antibody. After washing, 100 μL of peroxidase-conjugated polyclonal antibody specific to mouse VEGF was added to each well and incubated for 2 h at room temperature. Following substrate and a 30 min incubation, the enzymatic reaction was stopped. The resulting yellow color was measured, with its intensity directly proportional to the amount of VEGF in the sample.

#### 4.9.2. Determination of COX-2 Concentration

The expression of COX-2 was determined using the Human/Mouse Total COX-2 ELISA kit (R&D Systems Europe, Ltd., UK), according to the manufacturer’s instructions. The method is based on a sandwich ELISA, where immobilized binding antibodies bind specifically to COX-2. After washing unbound substances, a biotinylated detection antibody specific for COX-2 was applied, followed by a streptavidin–HRP conjugate. Absorbance was measured at 450 nm using a microplate reader (Labsystems iEMS Reader MF), with wavelength correction set at 540 nm. The intensity of the resulting yellow color is proportional to the amount of COX-2 initially bound. The COX-2 standard (120 ng/mL) provided in the kit was diluted with sample/standard dilution buffer in the concentration range of 0 to 10,000 pg/mL.

#### 4.9.3. Determination of MMP-2 and MMP-9 Concentrations

Levels of MMP-2 and MMP-9 were determined using the Mouse MMP-2 ELISA Kit and Mouse MMP-9 ELISA Kit (Chongqing Biospes Co., Ltd., Chongqing, China) according to the manufacturer’s protocols. These assays are based on the sandwich ELISA technique. Absorbance was measured at 450 nm using a Bio-Rad microplate reader Model 550 (Bio-Rad Laboratories, Inc. Hercules, CA, USA) to quantify color intensity. Standards of 10,000 pg/mL for MMP-2 or MMP-9 were prepared using the sample/standard dilution buffer to obtain final concentration range from 0 to 5000 pg/mL. The concentration of each sample was calculated from the standard curve based on the linear regression equations correlating absorbance and standard concentrations of MMP-2 or MMP-9.

#### 4.9.4. Cytokine Analysis

The levels of mouse Th1/Th2/Th17 cytokines were determined using the Mouse Th1/Th2/Th17 Cytokines “Multi-Analyte ELISArray Kit”, manufactured (QIAGEN Gmbh, Hilden, Germany) according to the manufacturer’s instructions. Cytokines were measured in ascites fluid. This assay detects the following cytokines: IL-2, IL-4, IL-5, IL-10, IL-12, IL-13, IL-17A, IL-23, IFN-γ and TNF-α, TGF-β1. Absorbance was measured at 450 nm with wavelength correction at 540 nm using a microplate reader (iEMS Reader MF, Labsystems, Helsinki, Finland). All samples were made in duplicate.

### 4.10. Comet Assay

We performed a standard alkaline comet assay on white blood cells and EAT cells [55]. A freshly prepared normal melting point (NMP) agarose solution was applied to microscope glass slide using a Pasteur pipette and covered with coverslip. After agarose polymerization at room temperature, the coverslip and agarose layer were removed. Next, 300 μL of 0.6% NMP agarose was added to the dried slides using a micropipette, covered with the coverslip, and kept on ice for 10 min. On top of this layer, we applied the next layer consisting of 100 μL of 0.5% LMP agarose mixed with 5 μL of either blood collected from the axillary plexus or EAT cells from the peritoneal fluid, and covered it again with a coverslip. Following 10 min incubation on ice, this layer was further covered with 100 μL of 0.5% LMP agarose, and the preparation was kept on ice again for 10 min. The coverslips were then removed, and the slides were immersed in lysis buffer (2.5 M NaCl, 1% Na-lauryl sarcosinate, 100 mM Na_2_EDTA, 10 mM Tris-HCl, 10% DMSO, and 1% Triton X-100, Sigma-Aldrich, St. Louis, MO, USA; DMSO (Kemika, Zagreb, Croatia)), pH = 10, for one hour at 4 °C. Following lysis, slides were transferred to the denaturation buffer (0.3 M NaOH, 1 mM Na_2_EDTA), pH = 13, and incubated at room temperature for 20 min. Following denaturation, the slides were transferred to a horizontal electrophoresis bath. Electrophoresis was performed in the same buffer as denaturation, at a current of 400 mA and a voltage of 25 V, for 20 min. After electrophoresis, the slides were washed three times for 5 min each in neutralizing buffer (0.4 M Tris-HCl), pH = 7.5. After the final wash, the slides were fixed in 96% ethanol for 7 min, and air-dried.

On the day of the analysis, the slides were stained with 100 μL ethidium bromide (20 μg/mL) for 10–12 min in the absence of light. The stained gels were briefly washed in Tris-HCl buffer, pH = 7, covered with coverslip, and kept in the dark for at least 15 min to stabilize the fluorescence before imaging. Stained preparations were analyzed using epifluorescence Leitz Wetzlar microscope and COHU High-Performance CCD Camera (Cohu Inc., CA, USA), MOD 4912-5000/0000 with an excitation filter of 515–560 nm. Tail length, percentage of DNA in the tail, and tail moment were quantified using the Comet Assay II image analysis program (Perceptive Instruments Ltd., Haverhill, UK.) For each experimental group, we analyzed 100 comets on each preparation (n = 4), i.e., a total of 400 cells from each experimental group.

### 4.11. Statitical Analysis

Statistical analyses were performed using the STATISTICA 12 program (StatSoft, Tulsa, OK, USA). Results are presented as mean ± standard error of the mean (Mean ± SE). Data was analyzed using the Kruskal–Wallis ANOVA test, followed by multiple comparisons of mean values across all groups. For VEGF, COX-2, MMP-2, MMP-9 and cytokine data, statistical significance was assessed using Student’s *t*-test. Descriptive statistics (mean, median, and standard error) were calculated for comet assay parameters: tail length, tail intensity, and tail moment. The extent of DNA damage in each sample was expressed as mean ± SE. To normalize the data distribution and equalize variances, a logarithmic transformation was applied. Multiple comparisons between groups were then performed on the log-transformed data. Post hoc analyses were conducted using the Scheffé test, with statistical significance set at *p* < 0.05.

## 5. Conclusions

In summary, our findings demonstrate that TA exerts anti-tumor and anti-angiogenic effects on EAT tumor through the regulation of TAM activity. TA may induce TAM repolarization and reduce VEGF/COX-2/MMP levels, and consequently inhibit angiogenesis and tumor growth. Inhibition of COX-2 with TA causes the loss of M2 macrophage characteristics of TAMs and enhances the antitumor immune response by increasing lymphocyte activation leading to favorable changes in the lymphocyte–monocyte ratio (LMR) and neutrophil–lymphocyte ratio (NLR) in peripheral blood. TA can induce DNA fragmentation in tumor and blood cells, indicating potential ROS-mediated cytotoxicity and induction of cells death by apoptosis and necrosis. These findings suggest that inhibition of TAMs by TA may be a promising strategy for the treatment of tumors and other conditions associated with angiogenesis. In conclusion, reduction in TAMs offers a promising strategy to modify the tumor microenvironment, reduce immune suppression, enhance immune responses in cancer, and improve patient outcomes. Due to the possible toxic effects of TA, further studies on normal cells are needed for the safe application of TA in food and pharmaceutical products.

## Figures and Tables

**Table 1:** Effect of TA on total number of cells, tumor growth inhibition, ascites volume and animal weight change (%) in mice bearing EAT.

Experimental Group ^a^	Total Number of Cells (×10^6^)	Min–Max Value (×10^6^)	Inhibition of Tumor Growth (%)	Volume of Ascitic Fluid	Min–Max Value	Reduction in Ascitic Fluid Volume (%)	Animal Weight Change (%)
**Control**	891.78 ± 76.32	672.00–1192.90		14.00 ± 0.43	12.10–15.60		24.40 ± 1.08
**TA 5 mg/kg**	223.98 ± 27.73 **	145.60–321.20	74.89	13.10 ± 0.45	11.20–14.60	6.43	22.17 ± 0.73
**TA 10 mg/kg**	223.14 ± 28.40 **	156.00–310.80	74.98	12.57 ± 0.85	11.00–14.80	10.2	21.30 ± 3.45

^a^ Mice were injected intraperitoneally (*ip*) with 2.5 × 10^6^ viable EAT cells and treated with TA at doses of 5 and 10 mg/kg *ip* during exponential tumor growth phase on days 5, 7, 9, and 11. The results are expressed as the mean value of each experimental group ± SE (*n* = 7 per group). ** *p* < 0.01 compared to control group. Abbreviations: EAT, Ehrlich ascites tumor; TA, tannic acid; SE, standard error.

**Table 2:** Differential analysis of ascites cells in peritoneal lavage fluid from EAT-bearing mice treated with tannic acid.

Experimental Group ^a^	Differential Analysis of Ascites Cells in Peritoneal Lavage Fluid (Mean ± SE)						
	Tumor Cells (%)	Lymphocytes (%)	Macrophages (%)	Neutrophils (%)	Basophils (%)	Eosinophils (%)	P/M
**Control**	73.75 ± 3.57	0.38 ± 0.24	11.00 ± 1.79	12.50 ± 3.34	2.00 ± 0.54	0.38 ± 0.24	1.31
**TA 5 mg/kg**	56.81 ± 4.12 *	0.44 ± 0.20	11.37 ± 1.92	31.31 ± 2.92 *	0.25 ± 0.13 *	0.25 ± 0.19	2.80
**TA 10 mg/kg**	55.31 ± 3.61 *	0.69 ± 0.38	21.38 ± 3.28 ** ^□^	21.44 ± 4.13	0.56 ± 0.33	0.63 ± 0.63	1.02

^a^ Mice were injected intraperitoneally (*ip*) with 2.5 × 10^6^ viable EAT cells and treated with TA at doses of 5 and 10 mg/kg *ip* during exponential tumor growth phase on days 5, 7, 9, and 11. The results are expressed as the mean value of each experimental group ± SE (*n* = 7 per group). * *p* < 0.05, ** *p* < 0.01 compared to control group; ^□^ *p* < 0.05 compared to 5 mg/kg TA group. Abbreviations: EAT, Ehrlich ascites tumor; TA, tannic acid; P/M, polymorphonuclear/mononuclear cells; SE, standard error.

**Table 3:** Absolute number of differential blood count and blood-based markers in EAT-bearing mice treated with tannic acid.

Experimental Group ^a^	Absolute Number of Differential Blood Count (Mean ± SE)								
	Total Leucocyte Number (×10^9^)	Lymphocytes (×10^9^)	Macrophages (×10^9^)	Neutrophils (×10^9^)	Basophils (×10^9^)	Eosinophils (×10^9^)	NLR	LMR	P/M
**Control**	5.48 ± 2.95	0.76 ± 0.15 (36.80%)	0.31 ± 0.09 (10.33%)	4.27 ± 2.92 (47.11%)	0.07 ± 0.01 (2.94%)	0.07 ± 0.02 (2.79%)	5.63	2.41	4.09
**TA 5 mg/kg**	4.79 ± 1.42	1.34 ± 0.23 (34.10%)	0.64 ± 0.26 (15.35%)	2.75 ± 1.32 (49.06%)	0.06 ± 0.01 (0.15%)	0.01 ± 0.00 (1.34%)	2.05	2.10	1.42
**TA 10 mg/kg**	9.17 ± 2.62 **	2.88 ± 0.53 ** ^□^ (42.23%)	0.37 ± 0.08 (5.54%)	5.78 ± 2.14 (50.40%)	0.06 ± 0.01 (0.74%)	0.07 ± 0.03 (1.08%)	2.00	7.82	1.82

^a^ Mice were injected intraperitoneally (*ip*) with 2.5 × 10^6^ viable EAT cells and treated with TA at doses of 5 and 10 mg/kg *ip* during exponential tumor growth phase on days 5, 7, 9, and 11. The results are expressed as the mean value of each experimental group ± SE (*n* = 7 per group). ** *p* < 0.01 compared to control group; □ *p* < 0.05 compared to 5 mg/kg TA group. Abbreviations: EAT, Ehrlich ascites tumor; TA, tannic acid; NLR, neutrophil-to-lymphocyte ratio; LMR, lymphocyte-to-monocyte ratio; P/M, polymorphonuclear/mononuclear cells; SE, standard error.

**Table 4:** Nitric oxide production and arginase 1 activity in the supernatant of spleen macrophages, ascites macrophages, and ascitic fluid from EAT-bearing mice treated with tannic acid.

Experimental Group ^a^	NO Levels in Supernatant of Macrophages or Ascitic Fluid (µM) (Mean ± SE)			Arg 1 Levels in Supernatant of Macrophages or Ascitic Fluid (µM) (Mean ± SE)		
	Spleen Macrophages	Ascites Macrophages	Ascites Fluid	Spleen Macrophages	Ascites Macrophages	Ascites Fluid
**Control**	15.87 ± 0.91	13.48 ± 0.75	20.26 ± 1.22	1027.41 ± 82.44	1048.57 ± 74.43	38,576.78 ± 2375.677
**TA 5 mg/kg**	15.86 ± 1.37	19.29 ± 2.32	33.33 ± 1.61 **	446.85 ± 72.64 **	590.19 ± 85.39 *	32,022.47 ± 874.395 *
**TA 10 mg/kg**	22.06 ± 0.96 ** ^□□^	18.71 ± 1.79	21.81 ± 2.65	273.10 ± 4.13 ***	767.65 ± 74.35 *	26,392.79 ± 482.421 **

^a^ Mice were injected intraperitoneally (*ip*) with 2.5 × 10^6^ viable EAT cells and treated with TA at doses of 5 and 10 mg/kg *ip* during exponential tumor growth phase on days 5, 7, 9, and 11. On day 13, mice were sacrificed, and supernatants from spleen macrophages, ascitic macrophages and ascitic fluid were collected. Levels of NO and Arg 1 were measured using ELISA. The results are expressed as the mean ± SE of the mean from three independent experiments per group; * *p* < 0.05, ** *p* < 0.01, *** *p* < 0.001 compared to control group; ^□□^ *p* < 0.01 compared to TA 5 mg/kg group. Abbreviations: EAT, Ehrlich ascites tumor; TA, tannic acid; SE, standard error.

**Table 5:** Tannic acid reduces VEGF secretion in the peritoneal cavity of mice bearing Ehrlich ascites tumor.

Experimental Group ^a^	Ascites Macrophages (pg/mL)	Ascites Fluid (pg/mL)	Tumor Cells (pg/mL)
**Control**	30.22 ± 3.58	1631.31 ± 39.7592	918.92 ± 110.247
**TA 5 mg/kg**	5.08 ± 1.02 ****	1430.44 ± 50.8062 *	344.44 ± 44.08 **
**TA 10 mg/kg**	7.66 ± 1.00 ****	1358.08 ± 128.439	453.50 ± 18.02 **

^a^ Mice were injected intraperitoneally (*ip*) with 2.5 × 10^6^ viable EAT cells and treated with TA at doses of 5 and 10 mg/kg *ip* during exponential tumor growth phase on days 5, 7, 9, and 11. Mice were sacrificed on day 13. The VEGF levels in ascites fluid and homogenates of tumor and macrophage cells from the peritoneal cavity were measured by ELISA kit. The results are expressed as the mean ± SE of the mean from three independent experiments per group; * *p* < 0.05, ** *p* < 0.01, **** *p* < 0.0001 compared to control group. Abbreviations: EAT, Ehrlich ascites tumor; TA, tannic acid; SE, standard error.

**Table 6:** Tannic acid treatment inhibits microvessel density (MVD) in the inner peritoneal lining of Ehrlich ascites tumor.

Experimental Group ^a^	Microvessel Density	Minimum	Maximum
**Control**	16.33 ± 4.14	9.00	23.33
**TA 5 mg/kg**	3.55 ± 0.48 *	2.66	4.33
**TA 10 mg/kg**	3.00 ± 0.51 *	2.33	4.00

^a^ Mice were injected intraperitoneally (*ip*) with 2.5 × 10^6^ viable EAT cells and treated with TA at doses of 5 and 10 mg/kg ip during exponential tumor growth phase on days 5, 7, 9, and 11. On day 13, mice were sacrificed, and after harvesting the EAT cells, the peritoneum was carefully open to examine angiogenesis in control and TA-treated tumor-bearing mice. The inner peritoneal lining was fixed in Bouin’s solution, photographed, and blood vessels were counted under the microscope. The results are expressed as the mean value ± SE (*n* = 7 per group). * *p* < 0.05 compared to control group. Abbreviations: EAT, Ehrlich ascites tumor; TA, tannic acid; SE, standard error.

**Table 7:** Tannic acid reduces COX-2 protein level in the peritoneal cavity of mice bearing Ehrlich ascites tumor.

Experimental Group ^a^	Ascites Macrophages (pg/mL)	Tumor Cells (pg/mL)
**Control**	124.66 ± 1.92	1873.00 ± 17.77
**TA 5 mg/kg**	18.00 ± 2.35 ****	851.00 ± 83.81 ***
**TA 10 mg/kg**	28.31 ± 5.49 ****	896.00 ± 176.57 **

^a^ Mice were injected intraperitoneally (*ip*) with 2.5 × 10^6^ viable EAT cells and treated with TA at doses of 5 and 10 mg/kg *ip* during exponential tumor growth phase on days 5, 7, 9, and 11. Mice were sacrificed on day 13. The COX-2 levels were measured by ELISA kit in homogenates of tumor or macrophage cells from the peritoneal cavity. The results are expressed as mean ± SE of the mean from three independent experiments; ** *p* < 0.01, *** *p* < 0.001, **** *p* < 0.0001 compared to control group. Abbreviations: EAT, Ehrlich ascites tumor; TA, tannic acid; COX-2, cyclooxygenase-2; SE, standard error.

**Table 8:** Tannic acid reduces MMP-2 and MMP-9 protein levels in the peritoneal cavity of mice bearing Ehrlich ascites tumor.

Experimental Group ^a^	Concentration of MMP-2 (ng/mL)	
	Ascites Macrophages	Tumor Cells
**Control**	5.13 ± 0.40	68.06 ± 4.46
**TA 5 mg/kg**	1.47 ± 0.25 ***	55.60 ± 2.35 *
**TA 10 mg/kg**	0.81 ± 0.27 ***	54.28 ± 3.48 *
	**Concentration of MMP-9 (ng/mL)**	
**Control**	6.97 ± 1.61	48.77 ± 3.7
**TA 5 mg/kg**	2.15 ± 1.83	40.31 ± 0.36 *
**TA 10 mg/kg**	3.58 ± 2.06	39.97 ± 0.78 *

^a^ Mice were injected intraperitoneally (*ip*) with 2.5 × 10^6^ viable EAT cells and treated with TA at doses of 5 and 10 mg/kg *ip* during exponential tumor growth phase on days 5, 7, 9, and 11. Mice were sacrificed on day 13. The MMP-2 and MMP-9 levels were measured by ELISA kit in homogenates of tumor or macrophage cells from the peritoneal cavity. The results are expressed as mean ± SE of the mean from three independent experiments; * *p* < 0.05, *** *p* < 0.001 compared to control group. Abbreviations: EAT, Ehrlich ascites tumor; TA, tannic acid; MMP-2, matrix metalloproteinase 2; MMP-9, matrix metalloproteinase 9; SE, standard error.

**Table 9:** Effect of tannic acid on comet assay parameters in whole blood and tumor cells.

Experimental Group ^a^	Comet Assay Parameters of Whole Blood Cells (Mean ± SE)					
	Tail Length (µm)	FC	Tail DNA %	FC	Tail Moment	FC
**Control**	15.83 ± 017		2.94 ± 0.33		0.38 ± 0.04	
**TA 5 mg/kg**	15.37 ± 1.42	0.97	8.15 ± 0.44 ***	2.77	0.87 ± 0.05 ***	2.29
**TA 10 mg/kg**	15.56 ± 2.62	0.98	5.6 ± 0.26 *** ^□□□^	1.90	0.67 ± 0.03 *** ^□□^	1.76
	**Comet assay parameters of tumor cells (Mean ± SE)**					
**Control**	17.60 ± 0.27		6.60 ± 0.57		0.97 ± 0.08	
**TA 5 mg/kg**	19.07 ± 0.23 ***	1.08	9.75 ± 0.51 ***	1.48	1.33 ± 0.07 **	1.37
**TA 10 mg/kg**	18.04 ± 0.24	1.02	9.94 ± 0.47 ***	1.51	1.28 ± 0.06 *	1.32

^a^ Mice were injected intraperitoneally (*ip*) with 2.5 × 10^6^ viable EAT cells and treated with TA at doses of 5 and 10 mg/kg *ip* during exponential tumor growth phase on days 5, 7, 9, and 11. Mice were sacrificed on day 13. The results are expressed as the mean ± SE. A total 400 comets per group were evaluated; * *p* < 0.05, ** *p* < 0.01, *** *p* < 0.001 compared to control group; ^□□^ *p* < 0.01, ^□□□^ *p* < 0.001 compared to TA 5 mg/kg group. Abbreviations: EAT, Ehrlich ascites tumor; TA, tannic acid; SE, standard error; FC, fold change (relative change in the parameter compared to the control).

**Table 10:** Effect of tannic acid on hematological parameters in mice bearing Ehrlich ascites tumor.

Groups ^a^	Hematological Parameters (Mean ± SE)									
	L (10^9^/L)	E (10^12^/L)	Hgb (g/L)	Hct (L/L)	MCV (fL)	MCH (pg)	MCHC (g/L)	RDW (%)	Plt (10^9^/L)	MPV (fL)
**EAT Control**	5.48 ± 2.95	7.08 ± 0.39	112.95 ± 5.20	0.37 ± 0.02	104.75 ± 0.46	31.95 ± 0.34	610.50 ± 5.32	14.23 ± 0.56	1262.50 ± 240.70	8.70 ± 3.33
**TA 5 mg/kg**	4.79 ± 1.42	7.47 ± 0.48	120.80 ± 7.46	0.40 ± 0.02	107.40 ± 2.16	32.40 ± 0.31	602.67 ± 7.51	14.70 ± 0.81	1676.00 ± 64.84	9.48 ± 3.91
**TA 10 mg/kg**	9.17 ± 2.62	8.42 ± 0.23	134.45 ± 4.15	0.44 ± 0.01	104.40 ± 0.47	31.95 ± 0.22	611.50 ± 2.50	14.95 ± 0.41	1325.50 ± 193.10	9.20 ± 3.94

^a^ Mice were injected intraperitoneally (ip) with 2.5 × 10^6^ viable EAT cells and treated with TA at doses of 5 and 10 mg/kg ip during the exponential tumor growth phase on days 5, 7, 9, and 11. On day 13, mice were sacrificed; liver, kidney and spleen were immediately collected and weighed individually. Data are presented as mean ± SE (*n* = 7 mice per group). Abbreviations: EAT, Ehrlich ascites tumor; TA, tannic acid; SE, standard error; E, erythrocytes; L, leukocytes; Hgb, hemoglobin; Hct, hematocrit; MCV, mean corpuscular volume; MCH, hemoglobin content in erythrocytes; MCHC, hemoglobin concentration in erythrocytes; RDW, erythrocyte size distribution; Plt, platelets; MPV, average platelet volume distribution.

**Table 11:** Effect of tannic acid on biochemical parameters in mice bearing Ehrlich ascites tumor.

Groups ^a^	Biochemical Parameters (Mean ± SE)							
	AST (U/L)	ALT (U/L)	LDH (U/L)	AMY (U/L)	CRP (mg/L)	GLU (mmol/L)	Urea (mmol/L)	TBIL (mmol/L)
**EAT Control**	421.25 ± 43.27	381.25 ± 109.02	12,672.50 ± 1865.48	3077.50 ± 142.16	0.38 ± 0.13	4.00 ± 0.58	5.50 ± 0.35	1.67 ± 1.25
**TA 5 mg/kg**	593.75 ± 68.29	665.25 ± 157.99	14,648.75 ± 1457.53	2516.25 ± 301.26	0.25 ± 0.14	4.25 ± 0.95	7.50 ± 0.74	2.31 ± 0.72
**TA 10 mg/kg**	475.00 ± 78.61	683.75 ± 193.20	10,385.00 ± 1787.02	3335.00 ± 223.55	0.38 ± 0.13	3.25 ± 0.25	5.75 ± 0.92	2.50 ± 1.44

^a^ Mice were injected intraperitoneally (ip) with 2.5 × 10^6^ viable EAT cells and treated with TA at doses of 5 and 10 mg/kg ip during the exponential tumor growth phase on days 5, 7, 9, and 11. On day 13, mice were sacrificed; liver, kidney and spleen were immediately collected and weighed individually. Data are presented as mean ± SE (n = 7 mice per group). Abbreviations: EAT, Ehrlich ascites tumor; TA, tannic acid; SE, standard error; AST, aspartate aminotransferase; ALT, alanine transaminase; LDH, lactate dehydrogenase; AMY, amylase, CRP, C-reactive protein; GLU, blood glucose, TBIL, total bilirubin.

## Data Availability

The original contributions generated for this study are included in the article; further inquiries can be directed to the corresponding author.

## Abbreviations

The following abbreviations are used in this manuscript:

Arg	Arginase
EAT	Ehrlich ascites tumor
COX-2	Cyclooxygenase -2
ECM	Extracellular matrix
ELISA	Enzyme-linked immunosorbent assay
FC	Fold change
IL	Interleukin
iNOS	Inducible nitric oxide synthase
LMR	Lymphocyte-to-monocyte ratio
MMP-2	Matrix metalloproteinase 2
MMP-9	Matrix metalloproteinase 9
MVD	Microvessel density
NK	Natural killer
NLR	Neutrophil-to-lymphocyte ratio
NO	Nitric oxide
PD-L1	Programmed death-ligand 1
P/M	Polymorphonuclear to mononuclear ratio
ROS	Reactive oxygen species
TA	Tannic acid
TAMs	Tumor-associated macrophages
TANs	Tumor-associated neutrophils
TGF-β	Tumor growth factor β
TNFα	Tumor necrosis factor α
VEGF	Vascular endothelial growth factor
